# A Randomized Control Trial of a Brief Self-Compassion Intervention for Perfectionism, Anxiety, Depression, and Body Image

**DOI:** 10.3389/fpsyg.2021.751294

**Published:** 2021-12-09

**Authors:** Vivian Woodfin, Helge Molde, Ingrid Dundas, Per-Einar Binder

**Affiliations:** Department of Clinical Psychology, Faculty of Psychology, University of Bergen, Bergen, Norway

**Keywords:** self-compassion, perfectionism, intervention, depression, anxiety, RCT, body image

## Abstract

**Objective:** Due to a rise in perfectionistic tendencies and growing concerns about the increase in mental health conditions among students this study aimed to examine the effects of a brief intervention in self-compassion on maladaptive perfectionism, anxiety, depression, and body image.

**Methods:** The intervention consisted of four seminars and a silent half-day retreat with short lectures and relevant experiential practices from Mindful Self-Compassion (MSC) and Mindfulness Based Stress Reduction (MBSR). This randomized wait-list control trial was pre-registered at Clinicaltrials.gov (ID: NCT03453437, Unique Protocol ID: UiBMSC2018). University students were randomly assigned to the intervention group and wait-list control group and filled out surveys weekly. A repeated measures analysis of variance (ANOVA) was used to compare the groups pre- and post-treatment. Mixed level modeling was used to analyze changes in all outcome measures over time.

**Results:** Eighty-nine participants completed the intervention. Results of the ANOVA showed significant post-intervention reductions in maladaptive perfectionistic tendencies and symptoms of depression and anxiety, in addition to increased self-compassion and improved body image in the intervention group as compared to the wait-list group. Mixed level modeling showed statistically significant changes in self-compassion, maladaptive perfectionism, adaptive perfectionism, anxiety, and depression but not body image. Only the mixed level modeling showed small but significant changes to adaptive perfectionism, also called strivings. Implications of different changes to maladaptive perfectionism than adaptive perfectionism are discussed.

## Introduction

Studies in western countries report a rise in several mental health conditions among university students. A longitudinal study by Oswalt et al. (2020) based on 454,029 students in the United States report significant increases in anxiety, ADHD, depression, insomnia, OCD, and panic attacks between 2009 and 2015. Duffy et al. (2019) used two large national datasets of 610,543 students and report a more than doubling of severe depression, non-suicidal self-injury, suicide plans, and suicide attempts between 2007 and 2018. This trend has also been observed in Norway, where more students report serious mental health problems than they did 8 years prior (Knapstad et al., 2018). In the most recent national survey, 29% Norwegian students reported having struggled with serious mental health problems (Knapstad et al., 2018). This trend is alarming, especially as it is unclear what is driving these changes. During the same time frame as researchers have observed the growing trend in mental health conditions, pressures on mental health services have also increased (Lipson et al., 2019). A study of 155,026 students found that rates of past year mental health service utilization on U.S. campuses increased from 19% in 2007 to 34% in 2017 (Lipson et al., 2019).

Perfectionism has recently been suggested to be a transdiagnostic process in mental illness and is linked to several mental health conditions, such as anxiety, depression, and eating disorders (Egan et al., 2011). Students also report increasing societal pressure to perform academically. A longitudinal study reports a 30-year trend of increases in perfectionism (Curran and Hill, 2019). Perfectionism is defined as having high personal standards and exaggerated self-criticism, doubts about actions and fear of making mistakes (Frost et al., 1990). It has been proposed that perfectionism consists of two factors (Stoeber and Otto, 2006; Stoeber, 2011; Woodfin et al., 2020). One factor, evaluative concerns, consisting of doubts about actions and fear of making mistakes and more often correlates to poor mental health outcomes such as depression, anxiety, and eating disorders, whilst the second factor, strivings, consists of high personal standards and is thought to be more adaptive, although this is still debated in the research field (Frost et al., 1990; Stoeber and Otto, 2006; Burgess et al., 2016; Woodfin et al., 2020).

Due to these rising trends in mental illness, more research is needed on ways in which to effectively prevent and treat psychological problems among young adults. In addition, due to perfectionism’s role in maintaining mental health problems, research is needed to understand whether interventions can effectively reduce perfectionism.

Whilst perfectionism consists of exaggerated self-criticism, doubts about actions and fear of making mistakes (Frost et al., 1990), self-compassion is one’s ability to treat oneself with kindness instead of criticism and judgment when experiencing failure and pain. As operationalized by Neff K. (2003), self-compassion consists of three diametrical components. The first is kindness as opposed to self-judgment which is described as treating oneself kindly, in particular when experiencing failure or suffering. The second component consists of common humanity as opposed to isolation, which means that one learns nobody is isolated or alone in making mistakes and experiencing distress, but rather that this is a shared human experience. Finally, the third component consists of mindfulness as opposed to overidentification which consists of experiencing distress without diminishing or exaggerating the importance of this (Neff K., 2003).

Previous studies on self-compassion interventions indicate that self-compassion courses can improve mental health over a short period of time, in large group settings and even online, and may therefore be both a time and cost-effective interventions to prevent and reduce mental health problems. In a multimethod project, including a quantitative multi-baseline randomized controlled trial (RCT), phenomenological interviews and a qualitative online survey, we found that a three-session self-compassion course gave gains in personal growth self-efficacy and healthy self-control, increases in self-compassion and reductions in anxiety and depression (Dundas et al., 2017). The participants experienced being more supportive and friendlier toward self, more aware of being too hard on oneself, feeling less alone when having painful feelings, more acceptance of painful feelings, and feeling more stable and peaceful, as the most important outcomes (Binder et al., 2019). Ferrari et al. (2019) conducted a meta-analysis to examine the effects of randomized controlled trials of 27 self-compassion interventions between 2010 and 2017. Results indicate that self-compassion interventions can lead to significant improvements in eating behavior, rumination, stress, depression, self-criticism, and anxiety. In addition, a systematic review of 28 studies indicate that self-compassion may be a potential buffer to both body and eating related outcomes (Braun et al., 2016). More studies are needed to both replicate these findings and to determine whether self-compassion interventions can have similar results in new populations and on new outcomes.

Also, lower self-compassion partially mediated the association between maladaptive perfectionism and depression in a college student sample, suggesting that maladaptive perfectionism may cause the negative effect on students mental health by lowering self-compassion (Mehr and Adams, 2016). In addition to being a suggested transdiagnostic process in mental health, perfectionism has also been described as a barrier to treatment because individuals who score higher on perfectionism have worse treatment outcome and are less likely to seek help (Blatt and Zuroff, 2002; Shannon et al., 2018). Perfectionism has long been considered a personality trait which can change in different contexts but remains relatively stable over a lifespan (Stoeber, 2018). However, perfectionism also correlates negatively with self-compassion. The more self-compassionate an individual is the less likely they are to score high on perfectionism (Neff K. D., 2003). This correlation is interesting because it has been unclear to what degree perfectionism can change over time, whilst self-compassion has shown to be malleable and can improve through self-compassion targeting interventions. Previous studies have shown that developing self-compassion through training can significantly reduce self-critical judgment (Beaumont et al., 2016).

The aim of this study was to examine the effects of a 3 week-long self-compassion course on perfectionistic tendencies, mental health and body image among Norwegian university level students. Our primary hypothesis was that a brief self-compassion course would be sufficient to increase self-compassion. A secondary aim of the study was to examine if the self-compassion course would also be sufficient to induce positive changes in perfectionism, body image and mental health. At baseline we expected low levels of maladaptive perfectionism and lower levels of depression, anxiety and body appreciation would be related to greater self-compassion. We expected higher levels of maladaptive perfectionism to be related to lower baseline self-compassion, higher levels of depression and anxiety, and lower levels of body appreciation. In summary, the aim of this study was to investigate the effects of a brief self-compassion course on perfectionistic tendencies, anxiety, depression and body image.

## Materials and Methods

### Participants

Eighty-nine of 221 student participants from two universities in Western Norway completed the self-compassion intervention. The mean age of participants was 27 (range 19–65) years. The sample consists of 21.6% men. The first baseline measure was also open to students who did not want to sign up for the course, as a result we measured drop-out rate from the second baseline. The first baseline measures were therefore only used for analyses of baseline correlations. Forty-five percent of students who filled out the survey more than once completed the course. *T*-tests showed no significant differences between these groups with the exception of levels of self-compassion. Participants who completed the course had higher baseline self-compassion means (*M* = 33.15) than the group that discontinued (*M* = 31.26). Participants attended the course anonymously, as a result only participants who let the course instructors know they would discontinue could be asked why they dropped out. The most common reasons for discontinuing were sickness, too many other engagements such as exams and family, moving, or having been randomized to the course with dates which did not fit their schedule.

### Procedure

The Norwegian Regional Committees for Medical and Health Research Ethics-North 2015/2211 approved this study and it was pre-registered at Clinicaltrials.gov. Participants were recruited in three waves, in the spring semester 2018, fall semester 2018, and spring semester 2019. All separate faculties were contacted in order to secure a more heterogenous population, however, the psychology faculty was excluded due to the instructors of the intervention’s affiliation to the psychology faculty. Each faculty was asked to assist with recruitment through the channels they most often used to contact students. For example, some faculties used newsletters whilst others contacted students by e-mail or through Facebook groups. The link was distributed by e-mail 2,381 times. Participants were given information in a short paragraph describing that we were conducting a research study on the effects of a brief self-compassion intervention. This brief description included a link to SurveyXact which directed interested students to an informed consent form. The informed consent included more details on the course and what the study entailed. This link was active for 2–7 days each semester. The first survey page provided potential participants with choices to consent to participate and sign up for the study or discontinue. Participants entered a lottery to win movie tickets each week they filled out the survey. Recruitment was limited to 90–110 students per semester (45–55 students per group) on a first come basis. Students who could not be included in the study were informed when the next course would be held. We expected up to 50% drop-out rate pre-intervention due to the accessibility of the online sign-up form. Participants were randomly assigned to the active intervention group or wait-list control group each semester. As a result, the course was held a total of six times for six separate groups, three experimental and three wait-list control groups. For the analyses, the three experimental groups were combined and the three wait-list control groups were combined. Each group filled out the surveys weekly including two baselines, a total of six times. In addition, the wait-list control group filled out a third baseline at the same time point as the experimental group completed the course.

### Intervention

The 3-week intervention consisted of a total of five sessions, four weekly seminars and a silent retreat, and was made up of self-compassion and mindfulness exercises from both the Mindful Self-Compassion (MSC) and Mindfulness Based Stress Reduction (MBSR) programs. Each weekly seminar lasted approximately 3 h with the exception of the silent retreat day which was held on a weekend and lasted 4 h. This extra retreat seminar is meant to allow students time to deepen their meditation practices without interruption. All the courses were led by the same two psychologists and teachers who had undergone teacher-training in MSC. An additional third MSC-teacher and psychologist was present to assist for the first two courses. Each session included brief 15-min lectures, followed by short guided exercises, group discussions, and guided experiential practices. The group was asked after the practice if anyone wanted to share and explore their experiences of each practice which in Mindful Self-Compassion is referred to as inquiry. In the experiential practices the task is to immerse oneself in the experience (for instance, to consider how they treat themselves and how they treat others in difficult situations), and then reflect upon these practices. After each weekly course students were given access to a new recorded audio of guided self-compassion exercises 10–20 min in length. Participants were encouraged to practice self-compassion daily until the following course.

The first session included a brief orientation of the course and a summary of all the sessions. Participants were introduced the structure of the course, and about “backdraft” and other possible challenges when working with self-compassion. Participants were asked to contact one of the two instructors always present if they experienced any difficulty during the course. Exercises during this first session included a meditation from MSC called “Why am I here.” Participants could explore first through meditation and reflection what had brought them to the course. Participants were then introduced the MSC exercise, “affectionate breathing.” This first session also included the writing and reflection exercise “How would you treat a friend.” The exercise asks students to reflect on how they treat themselves compared to how they would treat a close friend in a situation in which they are struggling, or they have made a mistake. The final exercise in session 1 was the “self-compassion break” which is designed to be used as an exercise to invoke self-kindness, common humanity and mindfulness in moments when one needs self-compassion. The exercises were followed by talking to another participant about the exercise, and a brief group inquiry by the course instructors. In addition to exercises and inquiry, the first session also included three brief psychoeducative lectures between 5 and 10 min long on compassion, self-compassion, self-compassion in every day life and a brief summary of self-compassion research.

The second session consisted of three exercises from MSC: “affectionate breathing,” “finding out what I really want,” and “soften, soothe, allow.” These exercises were new to participants and inquiry was used to gauge how participants were responding and allow participants to reflect on these experiences. Between these exercises we held two 15-min psychoeducative lectures on mindfulness: how to practice kind awareness, and how to accept and respond to difficult emotions, such as shame.

The third session’s theme was perfectionism and core values. In addition to repeating core meditation from session one and two, the third session introduced a new meditation from the MSC program called “loving-kindness for ourselves” and a core value exercise called “living with a vow” which allowed participants to reflect on which values are important to them. Between these meditations we held two brief lectures on perfectionism and core values.

The retreat day was held in silence so that participants could focus more in depth on their own experiences. However, participants were encouraged to speak to instructors if they encountered any difficulty. The retreat was held on campus on a weekend. Only exercises and no lectures were included on this day. Due to the length of the half-day silent retreat, we incorporated several movement exercises to lessen the strain of sitting for extended periods of time. The exercises included from MSC on this day were sense and savor walk, affectionate breathing, savoring food, soles of the feet, loving-kindness for ourselves, compassionate movement, and giving and receiving compassion. In addition, the MBSR exercise compassionate body scan was included. Participants had previous experience with all the MSC meditations from previous sessions with the exception of soles of the feet and savoring food.

The final session included the MSC meditations giving and receiving compassion, awareness of positive feelings and practicing gratitude. Like previous sessions, instructors held inquiry between each exercise, and brief lectures. The topics for the final session lectures were mindfulness and compassion; joy, gratitude and positive emotions; and compassion for oneself and others. Participants were also asked to reflect on which exercises had made the largest impact on them, and reflect on how self-compassion might be incorporated in their daily life going forward.

### Outcomes

#### Self-Compassion Scale- Short

The short version of the self-compassion scale consists of 12 of the original 26 items scored on a 5 point Likert scale from 1 (almost never) to 5 (almost always). The short version also includes both positive aspects of self-compassion, self-kindness and common humanity, and items from the negative subscales, which measure self-judgment, over-identification and isolation, and has shown a near-perfect correlation with the original scale (Raes et al., 2011). However, the original 26-item version is recommended for analyses at the subscale-level. The original self-compassion scale has been translated to Norwegian and with reportedly good psychometric properties in this population (Dundas et al., 2016).

#### Frost Multidimensional Perfectionism Scale-Brief

Frost Multidimensional Perfectionism Scale-Brief (FMPS-B) was used to measure perfectionistic tendencies (Frost et al., 1990; Burgess et al., 2016). The scale has eight items, four of which represent evaluative concerns and the remaining four represent strivings. Items are rated on a Likert scale from 1 (strongly disagree) to 5 (strongly agree). The original Frost multidimensional perfectionism scale consists of 35 items. The items included in the FMPS-B are items from 3 of the 5 the original subscales: concern over mistakes, doubts about actions, and personal standards. The scale was translated and validated with a Norwegian sample (Woodfin et al., 2020). The scale’s internal consistency (Cronbach’s alpha) in this study was 0.83. The validation study indicated that the FMPS-B consist of two factors consistent with previous literature on perfectionism which divided perfectionism into strivings and evaluative concerns. In the brief scale, strivings consists of items from the previous subscale personal standards and the factor evaluative concerns consists of items from the previous subscales concern over mistakes and doubts about actions (Frost et al., 1990; Burgess et al., 2016).

#### Body Appreciation Scale

The Body Appreciation Scale (BAS) consists of 13 items which assess body image on a Likert scale from 1 (never) to 5 (always). A higher score indicates higher body image appreciation the scale is widely used, and has acceptable reliability and validity (Avalos et al., 2005).

#### State-Trait Anxiety Inventory

The 20-item trait subscale of the State-Trait Anxiety Inventory (STAI) was used to measure symptoms of anxiety (Spielberger et al., 1983). The subscale’s 20 items are rated on a Likert scale from 1 (almost never) to 4 (almost always) and reflect general feelings of anxiety. The scale was previously validated in a Norwegian sample (Håseth et al., 1990) and had an internal consistency (Cronbach’s alpha) of 0.90 in our study (Woodfin et al., 2020).

#### Major Depression Inventory

Major Depression Inventory (MDI) was used to measure symptoms of depression over the past 2 weeks. The MDI consists of 13 items on a Likert scale from 1 (not at all) to 6 (all of the time) (Bech et al., 2001). There are two pairs of items in which only the highest score of each pair is used for the total score. The scale was previously validated in Danish (Olsen et al., 2003) and translated to Norwegian (Dundas et al., 2016). In this study, the instrument had an internal consistency (Cronbach’s alpha) of 0.88 (Woodfin et al., 2020).

### Statistical Analyses

We used a repeated measures analysis of variance (ANOVA) in order to test within and between differences of the control group and active treatment group. We expected there to be no significant differences between groups at the baseline measures when neither group had undergone the intervention, but significant differences between the groups at time 6, which was pre-treatment for the wait-list group and post-treatment for the active treatment group. Mixed level modeling was used in order to analyze if outcome measures changed significantly over time. For the mixed level modeling analysis, both groups were combined for more power. *A priori* power analysis was conducted to test the differences within and between two independent group means (mixed ANOVA) with a small to medium effect size (Cohen’s *d* = 0.30), and an alpha of 0.05. Results showed that the analysis required a sample of 90 participants to achieve a power of 0.80.

## Results

Baseline correlations showed that self-compassion correlated negatively with depression [*N*(353), *r* = −0.55, *p* < 0.01], anxiety [*N*(353), *r* = −0.72, *p* < 0.01] and positively with body appreciation [*N*(353), *r* = 0.59, *p* < 0.01], meaning students who scored higher on self-compassion at baseline had lower scores depression, anxiety and higher body appreciation.

The repeated measures ANOVA was used to compare the control group and active treatment group at both baseline times and after the treatment group had completed the course compared to the control group, which had not. A series of group (2) by time (3) repeated measures ANOVAs showed that there were significant differences between the control group and the active treatment group in levels of self-compassion, evaluative concerns, anxiety, depression, and body image at the time when only the active group had completed the self-compassion course. Self-compassion [*F*(1,84) = 11.10, *p* < 0.001, η*p2* = 0.12] and body image [*F*(1,84) = 6.32, *p* < 0.05, η*p2* = 0.07] increased significantly. Whilst anxiety [*F*(1,84) = 8.10, *p* < 0.01, η*p2* = 0.09], depression [*F*(1,84) = 7.20, *p* < 0.01, η*p2* = 0.08], evaluative concerns [*F*(1,84) = 5.15, *p* < 0.05, η*p2* = 0.06] decreased significantly. The only measure in which the groups showed no significant difference was perfectionistic strivings [*F*(1,84) = 0.14, *p* > 0.05, η*p2* = 0.00]. Simple main effects analysis using estimated marginal means with Sidak correction indicated no significant differences between groups at baseline. There were significant mean differences between groups at time 3 for anxiety [−6.94 (95% CI, −11.80–2.09) *p* < 0.01], depression [−5.85 (95% CI, −10.18- −1.52) *p* < 0.01], evaluative concerns [−2.14 (95% CI, −4.02- −0.26) *p* < 0.05], and body image [4.64 (95% CI, 0.97–8.32) *p* < 0.05]. Univariate tests of the estimated marginal means to test for effect of cohort indicated no significant differences between groups prior to the intervention. Pairwise comparisons of baseline times, indicated no significant changes for anxiety, depression, evaluative concerns, strivings, nor body image between time 1 to 2. However, there were significant changes between baseline 1 and 2 in self-compassion [1.07 (95% CI, 0.03–2.10) *p* < 0.05]. This means that students improved slightly in levels of self-compassion prior to the commencement of the course. Summarizing, the intervention seemed to have induced positive changes in all outcome variables but not significantly affected perfectionistic strivings.

A multi-level level modeling analysis of six measurement times of both groups combined after both groups had received the intervention, replicated the findings that there were statistically significant changes over time for self-compassion, evaluative concerns, anxiety, and depression. Self-compassion increased significantly over time (*B* = 1.47, *p* < 0.001). Evaluative concerns decreased significantly over time (*B* = −0.60, *p* < 0.001). Anxiety decreased significantly over time (*B* = −1.58, *p* < 0.001). And depression decreased significantly over time (*B* = −0.98, *p* < 0.001). However, unlike the results of the ANOVA, in this analysis strivings decreased significantly over time (*B* = −0.20, *p* < 0.001), whilst body image did not decrease significantly (*B* = −0.35, *p* > 0.05). In addition, there were significant differences in evaluative concerns explained by age (*B* = −0.11, *p* < 0.05) and anxiety explained by age (*B* = −0.33, *p* < 0.05) (see Table 1).

**TABLE 1:** Mixed level model of changes in mental health outcomes over time.

	Self-compassion	Depression	Anxiety	Evaluative concerns	Strivings	Body appreciation
(Intercept)	31.27***	21.46***	55.01***	13.55***	13.74***	26.79***
	(0.89)	(1.23)	(1.42)	(0.55)	(0.46)	(1.21)
c.age	0.09	−0.28*	−0.33*	−0.11*	–0.01	–0.03
	(0.10)	(0.12)	(0.15)	(0.05)	(0.05)	(0.08)
male	0.13	–1.53	–1.03	–0.73	–0.10	–0.46
	(1.68)	(2.14)	(2.65)	(0.97)	(0.95)	(1.38)
time	1.47***	−0.98***	−1.58***	−0.60***	−0.20***	–0.35
	(0.15)	(0.19)	(0.20)	(0.08)	(0.05)	(0.29)
AIC	2923.58	3291.52	3362.59	2226.76	2119.40	3670.22
BIC	2961.40	3329.35	3400.42	2264.58	2157.22	3707.97
Log likelihood	–1452.79	–1636.76	–1672.30	–1104.38	–1050.70	–1826.11

***p < 0.001;

*p < 0.05.

Summarizing, the analyses resulted in some mixed findings. In the multi-level modeling analysis perfectionistic strivings saw a significant reduction. However, this reduction was smaller than for evaluative concerns, and there were no significant differences in strivings in the ANOVA comparing the intervention group with the waitlist group. In addition, the analyses gave differing results for body appreciation, in that body appreciation increased after the intervention when comparing the groups in the ANOVA but not in the multi-level level modeling analysis which measured changes over 6 time points.

## Discussion

Results from this study indicate that self-compassion can increase through a brief 3-week mindfulness and self-compassion based intervention in a university setting. Increases in self-compassion were accompanied by decreases in anxiety and depression over time. These results support the meta-analysis conducted by Ferrari et al. (2019) which compiled and studied the effects of 27 self-compassion studies between 2010 and 2017. They observed significant changes in stress, depression, self-criticism, and anxiety.

Our results on changes in body image were mixed. When the intervention group was compared with the control group, significant positive changes were seen. However, when both groups had received the intervention and were combined in the multilevel analysis, this change did not reach significance. This contrasts to studies by Albertson et al. (2015) and Ferrari et al. (2019). In Ferrari’s and colleagues’ meta-analysis (2019) the largest effects of the self-compassion studies were seen for eating behavior and rumination. Furthermore, a self-compassion intervention study by Albertson et al. (2015) found significantly greater reductions in body dissatisfaction, body shame and contingent self-worth based on appearance in the active treatment group as opposed to their control group.

The differences between these results and our more inconsistent results, might be related to the fact that our intervention was not directed toward body dissatisfaction, nor did our sample show high levels of body dissatisfaction at baseline. As compared with baseline values in a female college student sample in the original study of Avalos et al. (2005), our baseline values were [*N*(365), *M* = 3.12], indicating that ours was a normal sample with regard to BAS. Perhaps changes in body appreciation are not to be expected in a sample which has not sought help for body related problems, especially not when the intervention was not specifically directed toward creating such changes.

The present results indicated that it is possible to decrease maladaptive perfectionism (evaluative concerns), but that adaptive perfectionism (perfectionistic strivings) may be less affected. Whilst evaluative concerns decreased significantly for the active group compared to the wait-list, perfectionistic strivings (adaptive perfectionism) did not. What is commonly referred to as adaptive perfectionism (perfectionistic strivings) remained stable. However, perfectionistic strivings did decrease slightly over the course of multiple time points as measured by mixed level modeling. Perfectionistic strivings consists of having high personal standards and expectations, whilst evaluative concerns consists of fear of making mistakes and doubts about one’s actions. As a result, strivings is to a greater degree consistent with one’s intrinsic interest in achieving high academic and other results, a factor which can remain more stable and consistent whilst individuals can simultaneously decrease fears of mistakes and doubts about actions. The implication of changing or reducing evaluative concerns but not strivings through an intervention targeting self-compassion, is that one factor of perfectionism may be more malleable than the more adaptive perfectionism factor. In addition, these changes in maladaptive perfectionism can occur irrespective of the stability in adaptive perfectionism. This indicates that students’ motivations to learn and achieve might not be reduced although their fear of failure might be. This is consistent with a prior study on the effect of a short self-compassion intervention in a similar student samples, where scores on the Personal Growth Initiative Scale (PGIS), increased, while depression and anxiety decreased (Dundas et al., 2017).

Although it is unclear why we found significantly higher levels of self-compassion at baseline among those who completed the course as opposed to those who dropped out, this may be partially explained by previous research and theory on outcome expectancy and fear of self-compassion. Patients’ expectations are considered an important common factor of psychotherapy outcome. Schulte (2008) defines outcome expectancy and developed a scale consisting of three main factors: hope of improvement, fear of change, and suitability. Previous research indicates that compassion for oneself may be experienced as threatening, even physiologically, for individuals who are highly self-critical, with insecure or avoidant attachment styles, or have experienced abuse, neglect or shame by caregivers (Rockliff et al., 2008; Liotti, 2010; Gilbert et al., 2011). Although self-compassion is an important component for treatment of mental health problems, fear of self-compassion may need to be addressed more specifically than this study was able to in a large anonymous group setting at a university.

It remains unclear how self-compassion acts as a mechanism of change for students. Prior studies have suggested that self-compassion may increase individual’ abilities to tolerate stressful feedback about one’s performance or person. Feedback on instances of failure may be a part of learning and can sometimes be harsh and humiliating in academic settings. In a series of studies with students, Leary et al. (2007) found that self-compassion may buffer reactions to situations that may involve failure, humiliation and rejection. Moreover, college students higher on self-compassion have been reported to have higher intrinsic motivation and lower performance motivation, meaning that they are motivated more by the wish to master new material, than the wish to win others’ approval for their performance (Neff et al., 2005).

### Strengths and Limitations

The strengths of this study are stringent randomization, a control group, and transparency through pre-registration and a detailed description of the intervention. This rigor and transparency allows others to replicate this study. In addition, this project addresses a gap in the literature, as no other study to date has used self-compassion interventions to address maladaptive perfectionism. However, this study also has several limitations. Our sample is limited to students from Western Norway, and primarily of female participants (78.4%), which may limit generalizability. In order to address the limitations of student populations we recruited from all but psychology faculties from two different universities. The use of wait-list control groups also poses some limitations to validity. The Hawthorne effect suggests that individuals will change their behavior when they are being observed/measured. In this study, participants could have for example accessed self-compassion literature and practices prior to beginning the course. Other studies also suggest the opposite, that participants could exaggerate symptoms in order to maintain their spot in the group, or because they will not act but rather wait to change. As a result, research suggests that wait-list control groups may have exaggerated effect sizes compared to no treatment or psychological placebo (Furukawa et al., 2014). In addition, due to drop-out we did not achieve enough power for the originally planned moderator analyses. Whilst accessibility to the course and anonymity were a major strengths of the intervention during recruitment, these also were a major limitation of our study, as many students signed up to participate online (55%) did not come to the course or dropped out. Students’ anonymity also limited our ability to follow up reasons why students chose not to attend or discontinued unless they contacted us. In addition, we did not measure for compliance of homework, meaning some participants may have been practicing meditations on their own more than others.

## Conclusion

The study adds to the literature on the positive effects of mindfulness and self-compassion based interventions, by showing that even a short 3-week mindfulness/self-compassion intervention can have positive effects on students perfectionism, as well as on anxiety and depression in a university setting. Interestingly, the intervention reduced negative perfectionism, more than it affected perfectionistic strivings. In accordance with other research we suggest that perfectionistic strivings may be an adaptive form of perfectionism (Stoeber and Otto, 2006), perhaps among students as well as athletes. This has implications for interventions aimed at counteracting the rise in several mental health conditions among students (Duffy et al., 2019; Oswalt et al., 2020). This study’s results implicate that being more self-compassionate may be possible without losing the positive aspects of perfectionistic strivings, such as wishing to do ones best.

## Data Availability Statement

The raw data supporting the conclusions of this article will be made available by the authors, without undue reservation.

## Ethics Statement

The studies involving human participants were reviewed and approved by the Norwegian Regional Committees for Medical and Health Research Ethics-North 2015/2211. The patients/participants provided their written informed consent to participate in this study.

## Author Contributions

VW and ID collected the data. P-EB and VW held the course. VW organized the database and wrote the first draft of the manuscript. HM and VW performed the statistical analyses. ID, HM, and P-EB wrote sections of the manuscript. All authors contributed to the conception and design of the study, contributed to the manuscript revision, read, and approved the submitted version.

## Conflict of Interest

The authors declare that the research was conducted in the absence of any commercial or financial relationships that could be construed as a potential conflict of interest.

## Publisher’s Note

All claims expressed in this article are solely those of the authors and do not necessarily represent those of their affiliated organizations, or those of the publisher, the editors and the reviewers. Any product that may be evaluated in this article, or claim that may be made by its manufacturer, is not guaranteed or endorsed by the publisher.
