# Supplementary material for: A Randomized Control Trial of a Brief Self-Compassion Intervention for Perfectionism, Anxiety, Depression, and Body Image
Source: Front Psychol. 2021 Dec 9;12:751294. doi: 10.3389/fpsyg.2021.751294 (PMC8695611; doi:10.3389/fpsyg.2021.751294)
Supplement: Supplementary file 1 [file Table_1.DOC]

**
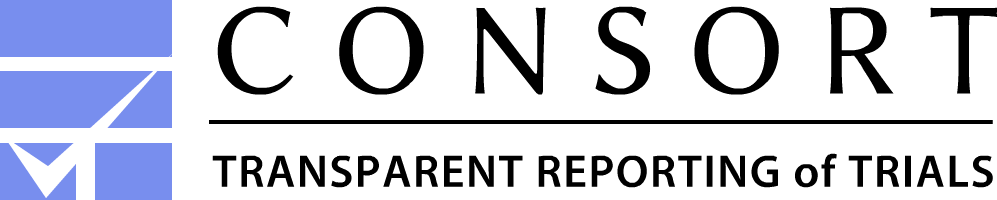
**

**CONSORT 2010 Flow Diagram**

**Allocation**

**Analysis**

**Follow-Up**

**Enrollment**

Assessed for eligibility (n= 336)

Excluded (n= 137)

♦  Not meeting inclusion criteria (n= 2)

♦  Declined to participate or other reasons, i.e. doubles, not completing survey or space in course (n= 135)

Analysed (n= 42)
♦ Excluded from analysis (give reasons) (n= 0)

Lost to follow-up/discontinued intervention (n= 52)

Allocated to intervention (n= 94)

♦ Received allocated intervention (n= 42)

♦ Did not receive allocated intervention (give reasons) (n= 52)

Lost to follow-up/ discontinued intervention (n= 58)

Allocated to intervention (n= 105)

♦ Received allocated intervention (n= 47)

♦ Did not receive allocated intervention (give reasons) (n= 58)

Analysed (n= 47)
♦ Excluded from analysis (give reasons) (n= 0)

Randomized (n= 199)
